# Expression of mRNA levels of HLA-DRA in relation to monocyte HLA-DR: a longitudinal sepsis study

**DOI:** 10.1186/cc14125

**Published:** 2015-03-16

**Authors:** SC Cajander, ET Tina, AB Bäckman, AM Magnuson, KS Strålin, BS Söderquist, JK Källman

**Affiliations:** 1 Örebro University, Örebro, Sweden

## Introduction

Decreased monocyte surface HLA-DR (mHLA-DR) measured by flow cytometry (FCM) is an independent marker of immunosuppression in sepsis. In a previous report we demonstrated that septic patients display a strong correlation between mHLA-DR and mRNA-levels of HLA-DRA in whole blood [1]. mRNA-based HLADR monitoring by PCR would improve the clinical usage and facilitate conduction of multicentre studies. The primary focus in this study was to evaluate the correlation between mHLA-DR and HLA-DRA at different time points during sepsis. In addition, we assessed the dynamic expression of both mHLA-DR and HLA-DRA, in relation to sepsis severity.

## Methods

Study patients (*n *= 54) were included at day 1 to 2 after hospital admission if blood cultures turned positive. Repeated sampling at days 1 to 2, 3, 7, 14 and 28 was performed. mHLA-DR was monitored by FCM and HLA-DRA by quantitative RT-PCR. Mixed models for longitudinal data were used after logarithmic transformation to calculate the interactional effects of time and severity on HLA-DR expression.

## Results

**Figure 1:**
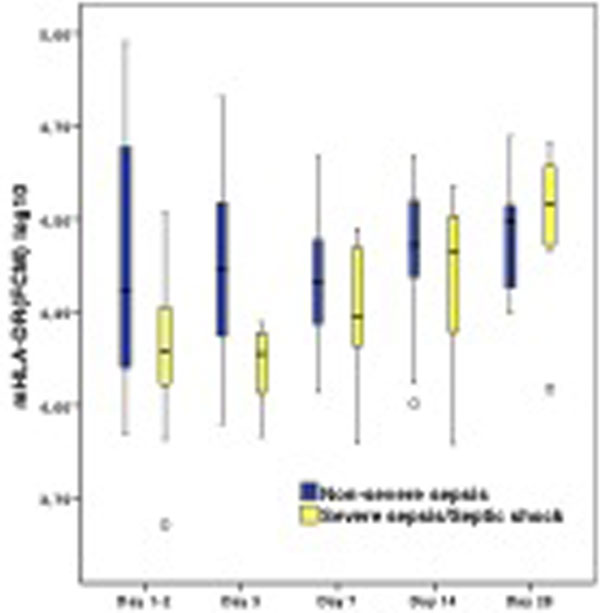
**Box plots of mHLA-DR, measured by flow cytometry**.

**Figure 2:**
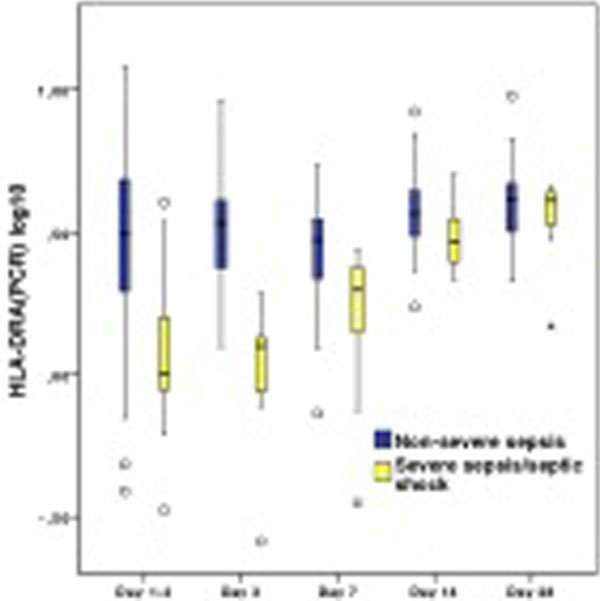
**Box plots of HLA-DRA, measured by qRT-PCR**.

Correlation between mHLA-DR(FCM) and HLA-DRA(PCR) at day 1 to 2 (*R *= 0.78) and day 14 (*R *= 0.27). Both HLA-DR markers increased linearly on a log scale over time. The linear association was significantly different between the severe (*n *= 16) and nonsevere septic patients (*n *= 38) when measuring either mHLA-DR(FCM) or HLA-DRA(PCR). By pairwise comparison of means between the two severity groups, at every time point, the differences between groups were shown to be significant at days 1 to 2 and 3 when monitoring mHLA-DR(FCM) and at days 1 to 2, 3 and 7 for HLA-DRA(PCR) (Figures 1 and 2).

## Conclusion

The correlation between flow cytometry and PCR-based HLA-DR monitoring is stronger in the early phase of sepsis. However, the linear associations over time, in relation to sepsis severity, display similar results for both HLA-DR markers. HLA-DRA(PCR) as a biomarker could be an alternative approach in monitoring immune status in sepsis but needs to be evaluated in relation to clinically relevant immunosuppression.
